# HOIL-1, an atypical E3 ligase that controls MyD88 signalling by forming ester bonds between ubiquitin and components of the Myddosome

**DOI:** 10.1016/j.jbior.2019.100666

**Published:** 2020-01

**Authors:** Philip Cohen, Ian R. Kelsall, Sambit K. Nanda, Jiazhen Zhang

**Affiliations:** MRC Protein Phosphorylation and Ubiquitylation Unit, University of Dundee, Scotland, United Kingdom

**Keywords:** TLR, LUBAC, HOIL-1, IRAK, Ubiquitylation, MyD88

## Abstract

Components of bacteria and viruses activate Toll-Like Receptors in host cells, triggering the formation of the Myddosome and a signalling network that culminates in the production and release of the inflammatory mediators required to combat pathogenic infection. The Myddosome initiates signalling by recruiting and activating five E3 ligases that generate hybrid ubiquitin chains and attach them to components of the Myddosome. These ubiquitin chains act as a scaffold for the recruitment and activation of ubiquitin-binding proteins, which include the “master” protein kinases TAK1 and IKKβ that drive inflammatory mediator production, as well as other proteins like ABIN1 and A20 that restrict activation of the network to prevent the overproduction of these substances that can lead to autoimmunity and organ damage. Here we review recent developments in our understanding of this network, focusing on the unexpected discovery that the E3 ligase HOIL-1 initiates the formation of hybrid ubiquitin chains by forming an ester bond between the first ubiquitin and the protein components of the Myddosome.

## 1 Introduction: similarities and differences between protein phosphorylation and ubiquitylation

I began to study the role of protein phosphorylation in cell regulation exactly 50 years ago to this day, when I joined Edmond Fischer's lab at the University of Washington, Seattle as a postdoctoral fellow. In those days just a few protein kinase activities had been identified and phosphorylation was thought to be a specialised control mechanism confined to the regulation of glycogen metabolism. My project focused on how the regulation of glycogen metabolism had evolved during vertebrate evolution (barely at all it transpired!) (Cohen et al., 1971, 1973). Now, 50 years later, we know that protein phosphorylation regulates almost all aspects of cell life, that almost every intracellular protein contains at least some covalently bound phosphate and that, to deal with this complexity, the human genome encodes over 500 protein kinases and nearly 200 protein phosphatases. Over 60 drugs that suppress protein kinase activities have been approved for clinical use since the first was approved in 2001 (Gleevec for the treatment of chronic myelogenous leukaemia), nearly all for the treatment of cancer.

In contrast, my love affair with protein ubiquitylation began only 12 years ago, after I switched my research focus to study the signal transduction pathways that control innate immunity. Discovered by Avram Hershko and Aaron Ciechanover in the late 1970's (Ciechanover et al., 1978) as a mechanism for degrading proteins, we now realize that, similar to phosphorylation, ubiquitylation also regulates most cellular processes. Ubiquitylation involves the formation of an isopeptide bond between the C-terminal carboxylate of ubiquitin and the ε-amino group of a lysine residue(s) on the target protein, and is catalysed by a relay of three enzymes, termed an E1 activating enzyme, an E2 conjugating enzyme and an E3 ligase. There are 2 E1s, about 40 E2s and roughly 600 E3s encoded by the human genome and over 100 deubiquitylases (DUBs) because, like phosphorylation, ubiquitylation is reversible. Similar to phosphorylation, ubiquitylation can occur at one or several sites and induce conformational changes that alter the biological functions of proteins.

The additional complexity of ubiquitylation arises from the subsequent attachment of ubiquitin molecules to one another, leading to the formation of protein-linked ubiquitin chains. Ubiquitin is a small protein comprising only 76 amino acid residues. There are seven lysine residues in the polypeptide chain, located at positions 6, 11, 27, 29, 33, 48 and 63, so that seven types of ubiquitin chain can be, and are, produced in cells. These ubiquitin chains adopt distinct conformations and so interact with different ubiquitin-binding proteins, which function to decode the ubiquitin signal (reviewed by Komander and Rape, 2012). Ubiquitin chains linked by lysine 48 or lysine 11 of ubiquitin (Finley, 2009; Jin et al., 2008) target the proteins to which they are attached to the 26S proteasome where they are degraded, the ubiquitin chains themselves being hydrolysed by DUBs and recycled for subsequent reuse. In contrast, other types of ubiquitin chain do not target proteins to the proteasome but modulate their functions in other ways.

**Fig. 1:**
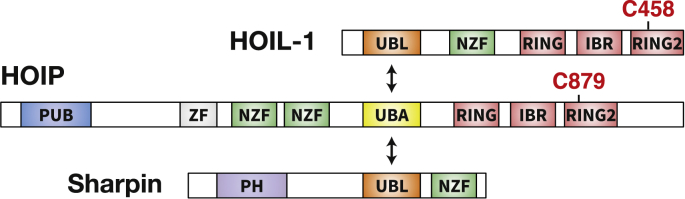
**Domain structures of the three components of LUBAC**. The LUBAC is formed from three subunits, HOIL-1, HOIP and Sharpin. HOIL-1 and HOIP are both members of the Really Interesting New Gene (RING)-Between-RING (RBR) subfamily of E3 ubiquitin ligases, with catalytic cysteines located at C458 (mouse HOIL-1) and C879 (mouse HOIP) in the C-terminal RING2 domain. The Ubiquitin Associated (UBA) domain of HOIP binds simultaneously to the Ubiquitin Like (UBL) domains of HOIL-1 and Sharpin. HOIP also contains a PUB (peptide:N-glycanase/UBA- or UBX-containing protein) domain, which interacts with the deubiquitylases Otulin and CYLD (cylindromatosis), the latter via the adaptor protein SPATA2 (spermatogenesis-associated protein 2).

A new twist to the field of ubiquitylation came with the discovery of the Linear Ubiquitin Assembly Complex (LUBAC) (Kirisako et al., 2006), which comprises the three proteins, HOIL-1 (haem-oxidised IRP2 ubiquitin ligase-1), HOIP (HOIL-1-interacting protein) and Sharpin (Fig. 1). HOIP, a member of the RING (Really Interesting New Gene)-Between-RING (RBR) family of E3 ligases was found to link the C-terminal carboxylate of ubiquitin to the α-amino group of the N-terminal methionine of another ubiquitin, forming Met1-linked ubiquitin (M1-Ub) chains (also called linear Ub chains). Therefore, the number of ubiquitin linkage types was eight and not seven. Here we describe the discovery of a 9th type of ubiquitin linkage and discuss its role in innate immune signalling.

## 2 Interplay between phosphorylation and ubiquitylation regulates innate immune signalling

**Fig. 2:**
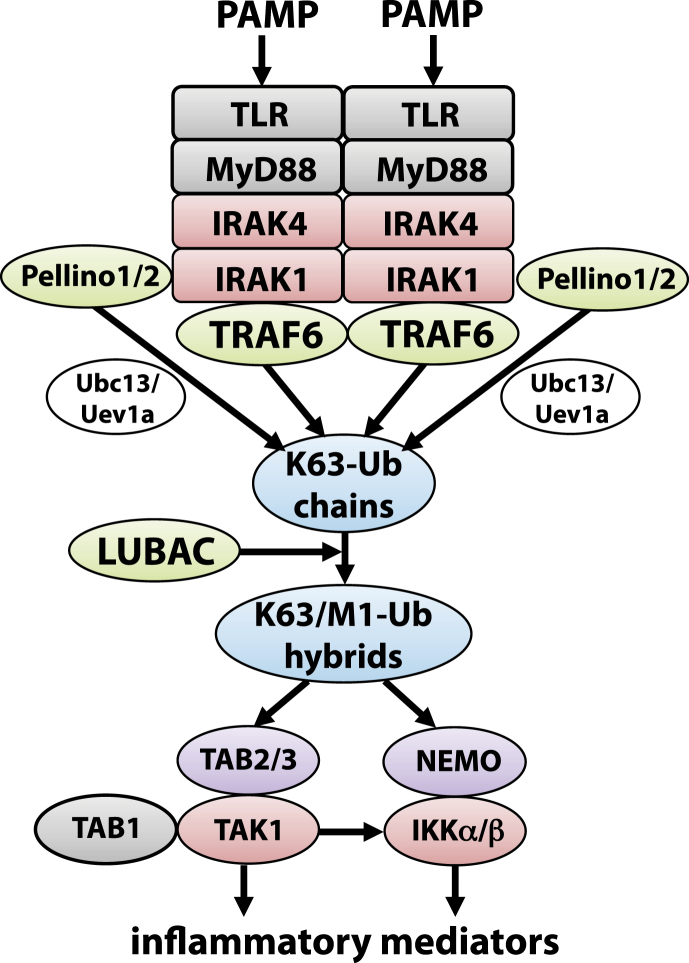
**TLR signalling triggers the formation of Lys63/Met1-linked hybrid ubiquitin chains and activation of the TAK1 and IKK complexes**. The interaction of PAMPs with TLRs triggers the formation of an oligomeric complex termed the Myddosome, comprising the adaptor protein MyD88 and protein kinases of the IRAK family. IRAKs 1 and 2 induce the activation of the E3 ligase TRAF6 while IRAK1 also phosphorylates and activates the E3 ligases Pellino 1 and 2. The three E3 ligases form productive complexes with the E2 conjugating complex Ubc13-Uev1a leading to the formation of Lys63-linked ubiquitin chains, which bind to the TAB2 and TAB3 subunits of the TAB1-TAK1-TAB2 and TAB1-TAK1-TAB3 complexes activating these protein kinases. The Lys63-linked ubiquitin chains also become a substrate for the HOIP-component of LUBAC, which is recruited into the signalling complex, forming Lys63/Met1-linked ubiquitin hybrids that recruit the canonical IKK complex. The interaction of Met1-Ub chains with the NEMO component of the IKK complex induces a conformational change that allows TAK1 to initiate the activation of the IKK complex by phosphorylating Ser176/177 of IKKα/β, the activation being completed by the IKKα/β-catalysed autophosphorylation of Ser180/181. Protein kinases are highlighted in red, E3 ligases in green, ubiquitin-binding proteins in purple and ubiquitin chains in blue.

Components of microbial pathogens, termed Pathogen-Associated Molecular Patterns (PAMPs), activate Pathogen Recognition Receptors (PRRs) in immune and other cells of the host, initiating signal transduction pathways that culminate in the production of pro-inflammatory cytokines and chemokines. These inflammatory mediators are secreted and mount the immune responses required to combat the invading pathogen. An important class of PRRs are the Toll-Like Receptors (TLRs), 10 of which are expressed in human cells. Nearly all TLRs, as well as the receptors for the Interleukin-1 (IL-1) family of cytokines, signal through the same adaptor protein, termed Myeloid Differentiating factor 88 (MyD88). The TLR/IL-1R-MyD88 interaction is followed by the recruitment of members of the Interleukin-Receptor-Associated-Kinase (IRAK) subfamily, forming an oligomeric complex, termed the Myddosome (Lin et al., 2010; Motshwene et al., 2009) (Fig. 2). The IRAK1 and IRAK2 molecules within this complex interact with TRAF6 (TNF-Receptor-Associated Factor 6), causing TRAF6 to adopt higher ordered structures that induce the activation of its E3 ubiquitin ligase function. A seminal finding was the discovery that the E2 conjugating complex Ubc13-Uev1a forms a productive complex with TRAF6, directing it to catalyse the formation of Lys63-linked ubiquitin (K63-Ub) chains, which then activate the “master” protein kinase TAK1 (TNF-Activated Kinase 1) (Deng et al., 2000; Wang et al., 2001). There are two hetero-trimeric TAK1 complexes, comprising TAB2 (TAK1-binding protein 2), TAK1 (the catalytic subunit) and TAB1 and TAB3-TAK1-TAB1 (Cheung et al., 2004). The K63-Ub chains bind to the NZF (Npl4 zinc finger) domains of TAB2 and TAB3, inducing conformational changes that activate the TAK1 complexes (Kanayama et al., 2004; Kulathu et al., 2009). TAK1 then initiates activation of both the canonical IκB kinase (IKK) complex (Fig. 2) and Mitogen-Activated Protein Kinase (MAPK) cascades that lead to the activation of p38 MAPKs and c-Jun N-terminal kinases (JNKs) (Zhang et al., 2014).

Ligands that activate TLRs and IL-1Rs fail to signal in TRAF6-deficient cells (Lomaga et al., 1999; Naito et al., 1999) and, for many years, the essential role of TRAF6 was thought to be the generation of K63-Ub chains. More recently, we found that the IL-1-stimulated formation of K63-Ub chains was unimpaired in TRAF6 KO HEK293 cells, and that IL-1 signalling could be restored by re-expressing the E3 ligase-inactive TRAF6[L74H] mutant. Similarly, the early phase of TLR signalling was reduced, but not abolished, in primary macrophages from knock-in mice expressing the TRAF6[L74H] mutant (Strickson et al., 2017). These findings implied that another K63-Ub chain-generating E3 ligase(s) had been activated during MyD88 signalling and that the essential role of TRAF6 was independent of its E3 ligase activity.

We found that the E3 ligase activity of TRAF6 was not essential because two other E3 ligases, Pellino 1 and Pellino 2, which are activated by IRAK1-catalysed phosphorylation events during MyD88 signalling (Goh et al., 2012; Ordureau et al., 2008; Smith et al., 2009), operate redundantly with TRAF6 in the generation of K63-Ub chains (Fig. 2) (Strickson et al., 2017). Consequently, primary macrophages from TRAF6[L74H] and wild-type mice produce and secrete similar amounts of the anti-inflammatory cytokine IL-10 during the first 2 h of TLR-MyD88 signalling. However, there is a drastic reduction in the production of pro-inflammatory cytokines, such as IL-6, and IL-12, and Chemokine (C–C) ligands, such as CCL3 and CCL4, which are mainly produced between four and 12 h after TLR-MyD88 signalling is initiated. This interesting difference appears to be explained by the disappearance of IRAK1 from macrophages 4 h after the initiation of signalling, leading to IRAK2 becoming rate-limiting for signalling (Pauls et al., 2013). IRAK2, appears to be a catalytically inactive pseudokinase, and should be unable to phosphorylate and so activate Pellino 1 and Pellino 2. However, it can still induce the oligomerisation and activation of TRAF6. Therefore, the TRAF6 E3 ligase activity does become essential when IRAK1 is absent or cannot be activated.

## 3 Role of Met1-linked ubiquitin chains in MyD88 signalling

A new development to this story began in 2009, with the discovery that the TNF-induced activation of the canonical IKK complex was impaired in embryonic fibroblasts (MEFs) from HOIL-1-deficient mice (Tokunaga et al., 2009). However, since the absence of HOIL-1 destabilises LUBAC and greatly reduces the expression of HOIP in murine cells, it was unclear at that time whether it was the loss of HOIL-1 and/or the loss of HOIP that was responsible for impaired IKK activation. We generated knock-in mice in which HOIP was replaced by the E3 ligase-inactive HOIP[C879S] mutant, a mutation that does not destabilise LUBAC and hence does not impair the expression of HOIL-1. We found that the IL-1-stimulated formation of M1-Ub chains was abolished (Emmerich et al., 2013) and activation of the canonical IKK complex was impaired in MEFs from the HOIP[C879S] knock-in mice (Zhang et al., 2014).

A few years earlier NEMO (NF-κB Essential Modifier), the regulatory subunit of the canonical IKK complex, had been shown to possess a ubiquitin-binding domain that interacted with K63-Ub oligomers (Ea et al., 2006; Wu et al., 2006), but it later became clear that this domain actually binds to M1-Ub dimers with 100-fold higher affinity than K63-Ub dimers (Lo et al., 2009; Rahighi et al., 2009). We found that IL-1-dependent IKK activation was reduced in embryonic fibroblasts from knock-in mice expressing the M1-Ub binding-defective NEMO[D311N] mutant (Zhang et al., 2014). Thus, both the formation of M1-Ub oligomers and their interaction with NEMO are required for the robust IL-1-dependent IKK activation in these cells (Emmerich et al., 2013; Zhang et al., 2014). In contrast, the IL-1-dependent activation of p38α MAPK and JNKs does not require M1-Ub chain formation and is therefore not reduced in MEFs from the E3 ligase-inactive HOIP[C879S] mutant (Zhang et al., 2014).

## 4 The formation and function of K63/M1-linked hybrid ubiquitin chains

We made the unexpected finding that the M1-Ub chains formed during IL-1 or TLR signalling were attached to K63-Ub chains, generating hybrid molecules containing both types of ubiquitin linkage (Emmerich et al., 2013). These observations implied that when LUBAC is recruited to the Myddosome, it initially selects K63-Ub oligomers as its preferred substrate thereby generating the K63/M1-Ub hybrids. Interestingly, the NZF domain of HOIP interacts specifically with K63-Ub oligomers, which may explain, at least in part, why the first M1-Ub linkage is made to a K63-Ub oligomer and not to monomeric ubiquitin. However, once the first hybrid linkage has been made, HOIP then switches to monomeric ubiquitin as its substrate, generating M1-Ub oligomers attached to K63-Ub oligomers. Precisely how this switch happens is unclear. Perhaps, the NZF domains of HOIL-1 have a role because, in contrast to the NZF domain of HOIP, they interact specifically with M1-Ub dimers (Sato et al., 2011). How LUBAC is actually recruited to IL-1/TLR signalling complexes is also unclear, but the expression of TRAF6 is needed because IL-1-dependent M1-Ub chain formation is greatly impaired in TRAF6 KO HEK293 cells and partially restored by re-expressing the E3 ligase-inactive TRAF6[L74H] mutant (Strickson et al., 2017).

What could be the advantages of forming K63/M1-Ub hybrids, rather than making the K63-Ub and M1-Ub chains as separate molecules? The formation of hybrid molecules is probably a device that enables the co-recruitment of proteins that bind to either type of ubiquitin linkage. For example, the selective binding of TAB2/3 to K63-Ub linkages and NEMO to M1-Ub linkages would be expected to lead to the co-recruitment of IKKs with their activator TAK1, facilitating TAK1-catalysed IKK activation (Fig. 2). We found that the activation of IKKβ is a two-step process, in which TAK1 first phosphorylates IKKβ at Ser177, followed by the IKKβ-catalysed autophosphorylation of Ser181. The IL-1-dependent phosphorylation of Ser177 is suppressed in HOIP[C879S] or NEMO[D311N] fibroblasts, demonstrating that the binding of M1-Ub chains to NEMO induces a conformational change that enables TAK1 to initiate the activation of IKKβ by phosphorylating Ser177 (Zhang et al., 2014).

The K63/M1-Ub hybrids also recruit proteins that restrict the strength of MyD88 signalling. MyD88 signalling is critical for defence against some microbial pathogens, but is a “double-edged sword” because the overproduction of inflammatory mediators, and/or failure to terminate inflammation once it has done its job, is a cause of inflammatory and autoimmune diseases, such as arthritis, asthma, colitis, fibrosis, lupus, psoriasis and sepsis. The strength of MyD88 signalling is therefore tightly controlled by a plethora of feedback control mechanisms and by the expression of inhibitors of the pathway (reviewed Cohen, 2014). Two of these, A20 and A20-binding inhibitor of NF-κB 1 (ABIN1), bind to the K63/M1-Ub hybrids as well as to one another. A20 contains a sequence of seven zinc fingers near its C-terminus, two of which interact with K63-Ub chains and M1-Ub chains, respectively (reviewed by Bosanac et al., 2010; Skaug et al., 2011), while ABIN1 possesses a ubiquitin-binding domain similar to that present in NEMO (Heyninck et al., 2003).

We generated knock-in mice that express the ubiquitin-binding-defective ABIN1[D485N] mutant (equivalent to the NEMO[D311N] mutant discussed earlier). TLR signalling was hyperactivated and pro-inflammatory cytokines overproduced in dendritic cells and B cells of the ABIN1[D485N] mice. The mice developed lupus spontaneously (Nanda et al., 2011), demonstrating the critical role of ubiquitin-binding to ABIN1 in restricting the strength of innate immune signalling. Interestingly, polymorphisms in ABIN1 that predispose to lupus and other autoimmune diseases have been identified in many different human populations (G'Sell et al., 2015). We found that every facet of the lupus phenotype could be prevented by crossing ABIN1[D485N] mice to MyD88 KO mice or to mice expressing kinase-inactive mutants of IRAK4 or IRAK1 (Nanda et al., 2016). These finding suggest that drugs inhibiting IRAK4 and/or IRAK1 may be useful for the treatment of some types of human lupus.

## 5 HOIL-1 regulates ubiquitin chain formation during TLR signalling by generating a 9th type of ubiquitin linkage

**Fig. 3:**
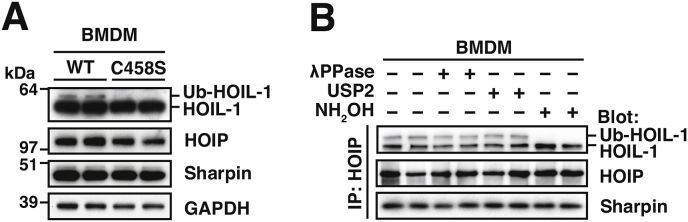
**A hydroxylamine-sensitive modification of HOIL-1**. (*A*) Immunoblots of extracts of bone-marrow-derived macrophages (BMDM) (20 μg protein) derived from wild-type (WT) and HOIL-1[C458S] knock-in mice. (*B*) LUBAC was immunoprecipitated with anti-HOIP from the extracts of WT macrophages, then treated with the protein phosphatase from bacteriophage lambda (λPPase), USP2 or hydroxylamine (NH_2_OH), followed by immunoblotting with the antibodies indicated. Adapted from results shown in Kelsall et al. (2019).

Like HOIP, the HOIL-1 component of LUBAC is also a member of the RBR subfamily of E3 ligases (Fig. 1). To identify its substrates and function *in vivo* we generated knock-in mice in which the catalytic cysteine of HOIL-1 (Cys458) was changed to serine, producing an E3 ligase-inactive mutant. In contrast to the equivalent HOIP[C879S] knock-in mouse (which dies at an early embryonic stage) (Emmerich et al., 2013), mice expressing the HOIL-1[C458S] mutant are born at normal Mendelian frequencies, are of normal size and weight and do not exhibit any obvious abnormalities, at least when kept for up to 6 months in the relatively sterile environment of the animal house (Kelsall et al., 2019). The three components of LUBAC are expressed at similar levels in macrophages from HOIL-1[C458S] or wild type mice, but we noticed that a minor, more slowly migrating component of HOIL-1 was not detectable in macrophages expressing HOIL-1[C458S], suggesting that it might be a mono-ubiquitylated form of HOIL-1 generated by autoubiquitylation (Fig. 3A). The more slowly migrating species of HOIL-1 was enriched within the LUBAC when it was immunoprecipitated from the extracts of wild type macrophages with anti-HOIP (Fig. 3B). To check whether the upper band of the HOIL-1 doublet was a monoubiquitylated HOIL-1, we incubated the immunoprecipitated LUBAC with USP2 (ubiquitin-specific proteinase 2), a broad-spectrum DUB that normally deubiquitylates proteins very efficiently. However, to our surprise, USP2 was ineffective and nor could the broad-spectrum protein phosphatase encoded by bacteriophage λ convert the upper to the lower band of the HOIL-1 doublet, indicating that the upper band was unlikely to be generated by phosphorylation (Fig. 3B).

Last year our colleagues at Dundee, Eric Pao and Satpal Virdee, identified MycBP2 as an E3 ligase able to conjugate the C-terminal carboxylate of ubiquitin to the hydroxyl side chain of a threonine, forming an ester bond (Pao et al., 2018). To investigate whether HOIL-1 might be another example of an ester-forming E3 ligase we incubated the immunoprecipitated LUBAC with hydroxylamine, a chemical that hydrolyses ester bonds but not isopeptide or peptide bonds. To our delight, hydroxylamine did indeed convert the upper to the lower band of the HOIL-1 doublet (Fig. 3B). We then used purified recombinant HOIL-1 to demonstrate that it was able to ubiquitylate itself at several serine and threonine residues, including Ser365 (Kelsall et al., 2019).

**Fig. 4:**
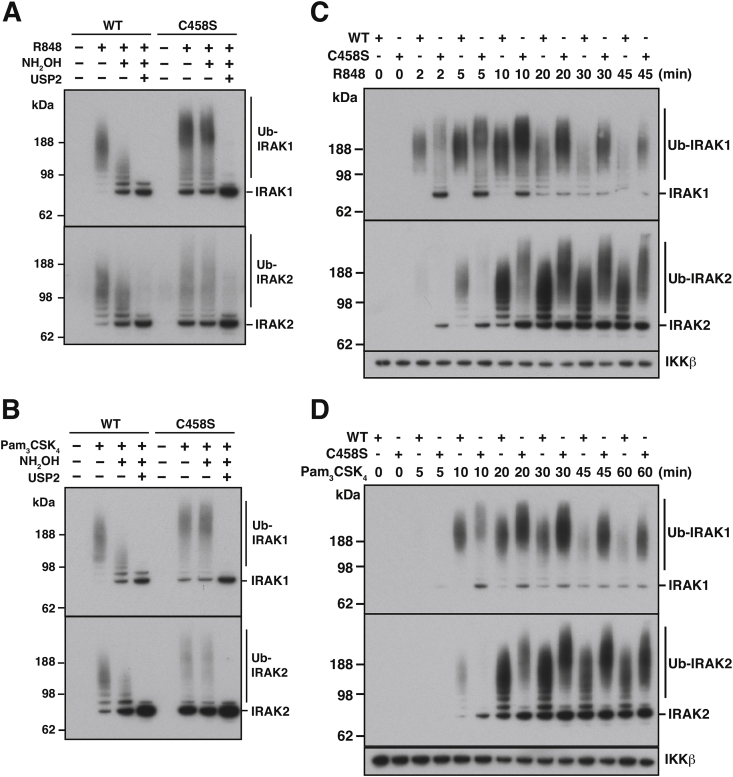
**The ubiquitin chains attached to IRAK1 and IRAK2 during TLR signalling are initiated by the formation of ester as well as isopeptide bonds**. (A, B) BMDM from WT or HOIL-1[C458S] knock-in mice were stimulated for 10 min with the TLR7 activating ligand 1 μg/ml R848 (A) or for 20 min with 1 μg/ml Pam_3_CSK_4_ an activator of the TLR1/2 heterodimer. The cells were lysed and ubiquitylated proteins captured from the cell extracts on Halo-NEMO beads and incubated for 30 min at 37 °C with λPPase. Following incubation for 60 min at pH 9.0 without (-) or with (+) 0.5 M hydroxylamine, the beads were incubated for a further 60 min at pH 7.5 without (-) or with (+) 1 μM USP2. Immunoblotting was performed with antibodies recognising IRAK1 or IRAK2. (C, D) Rate of formation of ubiquitylated IRAK1 and IRAK2 during TLR signalling. BMDM from WT or HOIL-1[C458S] mice were stimulated with 1 μg/ml R848 or 1 μg/ml Pam_3_CSK_4_ for the times indicated, lysed and ubiquitylated IRAK1 and IRAK2 captured from the cell extracts and treated with λPPase as in A, B. Phosphatase treatment was followed by immunoblotting with antibodies recognising IRAK1, IRAK2 and IKKβ (the latter acting as a loading control that binds to NEMO in a ubiquitin-independent manner). The figure is adapted from results presented in Kelsall et al. (2019).

Why does LUBAC contain two very different E3 ligase activities, HOIP and HOIL-1, within the same complex? We reasoned that this arrangement permits the co-recruitment of both E3 ligases to the same subcellular locations, suggesting that they might share common substrates. The K63/M1-Ub hybrids formed during IL-1 or TLR signalling are attached to a number of proteins, most notably the components of the Myddosome (Emmerich et al., 2013). We found that the ubiquitylated forms of IRAK1 and IRAK2 produced in macrophages during the first hour of TLR signalling contained hydroxylamine sensitive bonds (Fig. 4A and B) (Kelsall et al., 2019). Strikingly, hydroxylamine converted some, but not all of the ubiquitylated species to the unmodified IRAK1 and IRAK2 proteins (Fig. 4A and B) (Kelsall et al., 2019). These experiments established that some, but not all, of the ubiquitin chains attached to these proteins are initiated by the formation of an ester bond. In contrast, although TLR signalling still induced the ubiquitylation of IRAK1 and IRAK2 in macrophages from HOIL-1[C458S] mice (Fig. 4A and B), and also MyD88 (Kelsall et al., 2019), these ubiquitylated species were resistant to hydroxylamine. These experiments demonstrated that the hydroxylamine-sensitive species of IRAK1, IRAK2 and MyD88 are formed by the action of HOIL-1.

## 6 Cooperation between the HOIL-1 and HOIP components of LUBAC in MyD88 signalling

**Fig. 5:**
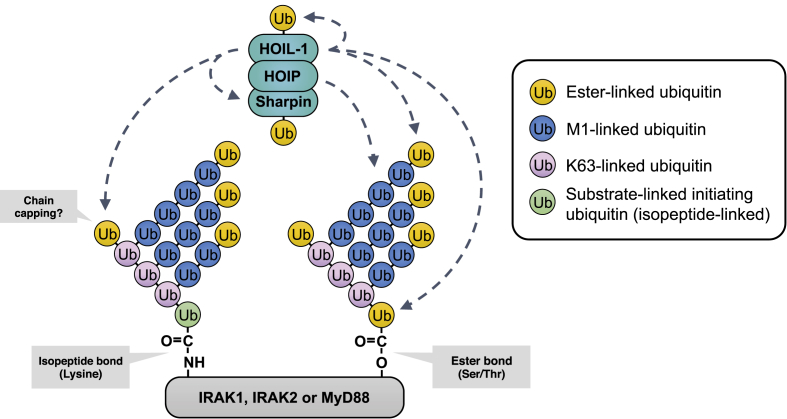
**Schematic of the linkage types present within the ubiquitin chains attached to components of the Myddosome**. Two types of Ub chain are attached to IRAK1, IRAK2 and MyD88. One is initiated by the attachment of ubiquitin to a serine or threonine residue(s) on these proteins and is catalysed by HOIL-1, while the other is initiated by the formation of an isopeptide bond to a lysine residue(s). Ubiquitin is also attached covalently to HOIL-1 and Sharpin by ester bonds. HOIL-1 can additionally catalyse the formation of ubiquitin dimers linked by an oxyester bond *in vitro*, but whether these linkages are present in the ubiquitin chains that become attached to IRAK1, IRAK2 and MyD88 during TLR signalling is not established. In the schematic we speculatively place such linkages at the terminal K63-Ub and M1-Ub linkages as a “capping” mechanism to explain why the ubiquitin chains that become attached to IRAK1 and IRAK2 are much larger in macrophages from mice expressing an E3 ligase-inactive mutant of HOIL-1 (Fig. 4C and D).

The LUBAC is remarkable in containing two atypical E3 ligase activities. One (HOIP) forms Met1-linked ubiquitin chains, while the other (HOIL-1) forms ester bonds between ubiquitin and the side chains of serine and threonine residues. Therefore the 8th and 9th types of ubiquitin linkage to be discovered are produced by different components of the same protein complex. HOIL-1 seems to be predominantly a monoubiquitylating E3 ligase that attaches the first ubiquitin to proteins, some of which are elongated and/or branched by the subsequent action of HOIP and other E3 ligases that catalyse the formation of K63-Ub linkages (Kelsall et al., 2019). The complex ubiquitin chains formed during TLR/MyD88 signalling therefore contain three ubiquitin linkage types, introduced by five E3 ligases (Fig. 2, Fig. 5).

## 7 HOIL-1-catalysed formation of ester-linked ubiquitin oligomers?

Intriguingly, HOIL-1 can monoubiquitylate ubiquitin *in vitro* forming unique ubiquitin dimers linked by Thr12 (Kelsall et al., 2019). Whether such ester bonds linking two ubiquitin molecules are formed in cells is unclear but, if they are, we speculate that one of their functions may be to limit the size that particular ubiquitin chains can attain. This would explain why the ubiquitin chains attached to IRAK1 and IRAK2 during TLR signalling are much larger in HOIL-1[C458S] macrophages than in wild type macrophages (Fig. 4, Fig. 5). Interestingly, interrogation of the cBioPortal for Cancer Genomics database (Cerami et al., 2012; Gao et al., 2013) reveals Thr12Ala and Thr12Ile mutations in the ubiquitin of patients suffering stomach adenocarcinoma and cutaneous melanoma respectively (cBioPortal for Cancer Genomics, https://www.cbioportal.org, accessed on 18 Sep 2019), raising the possibility that inability to form ester-linked ubiquitin may predispose to cancer. The phosphorylation of ubiquitin at Thr12 has also been detected in several cell lines (Kettenbach et al., 2011; Lee et al., 2009; Zhou et al., 2013), providing another way in which the HOIL-1-catalysed formation of ubiquitin dimers could be prevented. It would be of interest to identify the protein kinase(s) that phosphorylate(s) Thr12.

## 8 What are the physiological consequences of the loss of HOIL-1 E3 ligase activity?

We have recently begun to investigate how the TLR/MyD88-dependent production of cytokines and chemokines is affected in macrophages from mice expressing the E3 ligase-inactive HOIL-1[C458S] mutant. Interestingly, initial results indicate that the early phase of TLR/MyD88 signalling leading to the production of immediate early genes and the anti-inflammatory cytokine IL-10 is little affected, but the production of several pro-inflammatory cytokines and chemokines during the late phase of TLR/MyD88 signalling from 4 to 12 h is markedly decreased. As discussed earlier in this article, IRAK2 becomes rate-limiting for TLR/MyD88 signalling during the late phase because IRAK1 disappears after the early phase. Moreover, the expression of IRAK2 increases considerably during the late phase. Initial results indicate that the ubiquitylation of IRAK2 increases during the late phase and that the majority of the IRAK2-linked ubiquitin chains are initiated by HOIL-1-catalysed ester linkages. The absence of IRAK2-linked ubiquitin chains in HOIL-1[C458S] macrophages may therefore underlie the decreased production of inflammatory mediators that occurs during this period. Since ester-linked ubiquitin chains remain attached to IRAK2 for many hours and are even increased during late phase TLR-signalling, these ester bonds may be more resistant to deubiquitylation than isopeptide-linked ubiquitin. This would be consistent with the observation that the ester-linked ubiquitin attached to HOIL-1 resists cleavage by USP2 (Fig. 3B), a DUB that hydrolyses isopeptide-linked ubiquitin efficiently. Therefore, initiating ubiquitin chains with HOIL-1-catalysed ester bonds may be a device used to extend the duration of MyD88 signalling. However, the details of the late phase of TLR/MyD88 signalling are relatively unexplored compared to the early phase, and HOIL-1 might well have other functions during the late phase that have yet to be discovered.

The reduced production of pro-inflammatory cytokines and chemokines, without a reduction in the anti-inflammatory cytokine IL-10 in macrophages from HOIL-1[C458S] mice, suggests that this E3 ligase may be an interesting target for the development of a novel anti-inflammatory drug. It will therefore be of great interest to investigate how other MyD88 signalling pathways triggered by different ligands in other cells are affected by the absence of the HOIL-1 E3 ligase. For example, the over-production of IL-13 and other cytokines induced by IL-33 in mast cells and type 2 innate lymphoid cells is a cause of asthma. IRAK3 has been reported to have a critical role in IL-33 signalling (Nechama et al., 2018) and it will be interesting to study whether this IRAK family member is a physiological substrate for HOIL-1 in this pathway.

In summary, the discovery that HOIL-1 is an ester-bond forming E3 ubiquitin ligase has revealed novel ways in which LUBAC regulates the innate immune system, and perhaps other biological processes in which LUBAC participates, such as Wnt signalling (Rivkin et al., 2013), protein quality control (van Well et al., 2019) and anti-apoptotic signalling in response to genotoxic agents (MacKay et al., 2014).

## Animal studies

Experiments using macrophages from mice that were used to generate the results shown in Fig. 3, Fig. 4 were approved by the University of Dundee Ethical Review Board under a UK Home Office project licence.

## Declaration of competing interest

None.
